# Prognostic impact of diabetes mellitus on hepatocellular carcinoma: Special emphasis from the BCLC perspective

**DOI:** 10.1371/journal.pone.0174333

**Published:** 2017-03-23

**Authors:** Yu-Wen Su, Po-Hong Liu, Chia-Yang Hsu, Yun-Hsuan Lee, Cheng-Yuan Hsia, Shu-Yein Ho, Ming-Chih Hou, Harn-Shen Chen, Teh-Ia Huo

**Affiliations:** 1 Departments of Medicine, Taipei Veterans General Hospital, Taipei, Taiwan; 2 Faculty of Medicine, National Yang-Ming University School of Medicine, Taipei, Taiwan; 3 Department of Internal Medicine, University of Nevada School of Medicine, Reno, Nevada, United States of America; 4 Departments of Surgery, Taipei Veterans General Hospital, Taipei, Taiwan; 5 Institute of Pharmacology, National Yang-Ming University School of Medicine, Taipei, Taiwan; Chang Gung Memorial Hospital Kaohsiung Branch, TAIWAN

## Abstract

**Background:**

Diabetes mellitus (DM) is associated with higher incidence and poorer prognosis of hepatocellular carcinoma (HCC). The influence of DM on patient survival in different HCC stages is not known.

**Methods:**

A prospective dataset of 3,182 HCC patients was collected between 2002 and 2014. Patients were divided into three groups according to BCLC stages (BCLC stage 0 and stage A, BCLC stage B, BCLC stage C and stage D). We compared the cumulative survival rate of diabetic and non-diabetic patients in different BCLC groups. The correlation between DM and overall survival was also analyzed by multivariate Cox regression model within each group.

**Results:**

DM is present in 25.2% of all patients. Diabetic patients had lower cumulative survival in BCLC stage 0 plus BCLC stage A group (log rank *p*<0.001), and BCLC stage B group (log rank *p* = 0.012), but not in BCLC stage C plus BCLC stage D group (log rank *p* = 0.132). Statistically significant differences in overall survival are found between diabetic and non-diabetic patients in BCLC stage 0 plus stage A group (adjusted hazard ratio [HR] = 1.45, 95% confidence interval [CI] 1.08–1.93, *p* = 0.013), and BCLC stage B (adjusted HR = 1.77, 95% CI 1.24–2.51, *p* = 0.002). In contrast, the survival difference is not seen in BCLC stage C plus stage D group (adjusted HR = 1.09, 95% CI 0.90–1.30, *p* = 0.387).

**Conclusions:**

DM is prevalent in HCC, and is associated with lower survival rate in HCC patients with BCLC stage 0 plus stage A and B, but not in those with BCLC stage C plus stage D.

## Introduction

Hepatocellular carcinoma (HCC) is the fifth common neoplasm in men and the seventh in women. It contributed to 745,000 deaths in year 2012 and was the second leading cause of cancer-related mortality worldwide.[1] Well-established risk factors for HCC include chronic hepatitis B virus (HBV) infection, chronic hepatitis C virus (HCV) infection, aflatoxin B1, and alcohol consumption.[2, 3] The pathogenic and prognostic roles of metabolic factors, such as diabetes mellitus (DM), metabolic syndrome, or obesity, had also been studied.[4–6] Epidemiologic studies have disclosed association between presence of DM and higher HCC incidence, suggesting that DM is an independent risk factor for development of HCC.[7–10]

In addition to its role in pathogenesis, DM may also be an important predictor of prognosis.[11, 12] Previous studies analyzing the relation between DM and HCC outcomes focused mainly on resectable or potentially curable diseases. However, the results were inconsistent.[13–18] DM seems to worsen HCC prognosis in some subgroups to a greater extent. Toyoda *et al* found that the presence of DM led to poorer prognosis only in patients with treatable diseases, and those with tumor size ≤ 3 cm in greatest dimension.[19] Wang *et al* reported lower overall and disease-free survival in DM patients with cirrhosis and HCC, but not in their non-cirrhotic counterparts.[20]

The Barcelona Clínic Liver Cancer (BCLC) classification is one of the most widely adapted classification systems for HCC. The BCLC system incorporates multiple factors, including tumor burden, liver functional reserve and performance status. With its ability to predict prognosis and guide treatment algorithm, the BCLC staging system is endorsed by the European Association for the Study of Liver (EASL) and American Association for the Study of Liver Diseases (AASLD) HCC management guidelines. BCLC system stratifies patients into several distinct prognostic groups. The association and prognostic impact of DM on HCC patients with different cancer stages remain unclear. In this study, we aim to explore the prognostic role of DM in different BCLC stages.

## Methods

### Patients

We have prospectively enrolled and retrospectively analyzed newly diagnosed HCC patients admitted to Taipei Veterans General Hospital during 2002 to 2014. Baseline characteristics, including underlying etiologies for HCC, biochemistry profile, tumor extent, vascular invasion, severity of cirrhosis, performance status, and diagnosis of DM were recorded. Patient follow-up was arranged every 3–6 months until death or dropout from the program. Survival was defined from the date of diagnosis to death or last follow-up. Those receiving liver transplantation were censored at the date of transplantation. The study complies with the standards of the Declaration of Helsinki and current ethical guidelines and was approved by the Institutional Review Board of the Taipei Veterans General Hospital (IRB protocol number 2014-03-007AC). Waiver of consent was obtained, and patient records/information was anonymized and de-identified prior to analysis.

### Diagnosis and definitions

The diagnosis of HCC was established in accordance with EASL and AASLD HCC management guidelines.[21, 22] BCLC staging information was obtained at the time of diagnosis. We defined vascular invasion as radiological evidence of tumor invasion to intrahepatic vasculatures, portal trunk or abdominal great vessels. DM was defined as a fasting plasma glucose of 126 mg/dl or greater on at least two separate occasions, plasma glucose of 200 mg/dl or greater 2 hours after a 75 g oral glucose tolerance test, a glycated hemoglobin (HbA_1C_) level > 6.5% for once, or any prescription of hypoglycemic agents.[23]

### Treatments

Once diagnosis was confirmed, patient data were reviewed at multi-disciplinary HCC board of Taipei Veterans General Hospital for treatment planning. We provided comprehensive information regarding risks and benefits of each treatment to patients. The final treatment modality taken was decided by shared-decision making between physicians and patients. Written informed consent was obtained prior to all management. Invasive therapies, including radiofrequency ablation, surgical resection, and transarterial chemoembolization were performed through standard procedures as previously reported.[24–26]

### Statistics

The cumulative survival rates of diabetic and non-diabetic patients among different BCLC stages were examined by the Kaplan-Meier methods with log-rank tests. Cox proportional hazards regression model was performed for hazard ratio evaluation. Prognostic factors that are probably associated with overall survival, including age, sex, severity and etiology of chronic liver diseases, biochemical laboratory parameters and tumoral status were included in the univariate survival analysis. Factors significant in the univariate analysis (*P* < 0.1) were introduced into the multivariate Cox model to determine independent predictors of prognosis. The proportional hazard assumption was assessed graphically before being analyzed with Cox model. We used two-tailed χ^2^ test to compare categorical data, and Mann-Whitney *U* test to evaluate continuous variables. Interaction between DM and other predictors were assessed using likelihood ratio tests comparing the final model and the final model with the interaction terms. Statistical analyses were conducted with IBM SPSS version 21 (IBM, NY) and SAS version 9.4 (SAS Institute, NC). Statistical significance was set as *P* value less than 0.05 in a two-tailed test.

## Results

### Patient characteristics

**Fig 1:**
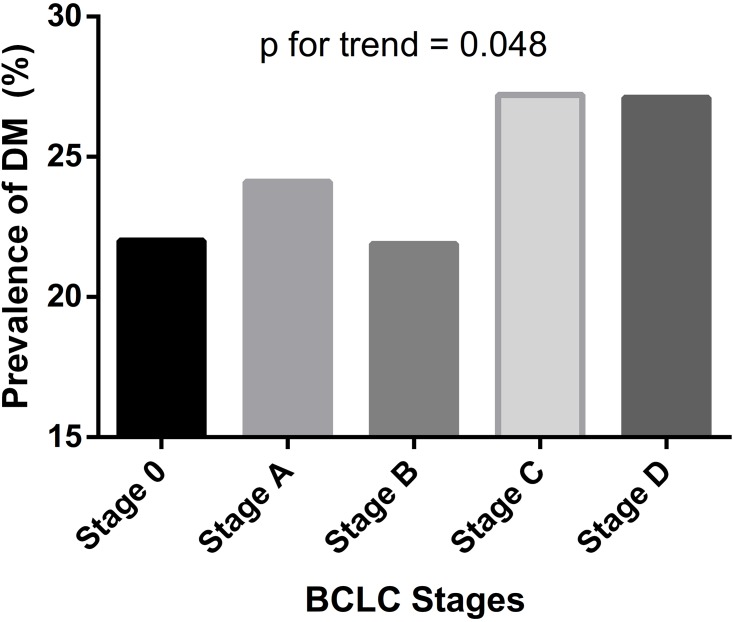
Prevalence of Diabetes Mellitus (DM) in different BCLC stages. A trend toward increasing DM prevalence in more advanced BCLC stages is seen at a marginal significance (p for trend = 0.048).

The median age of the study patients is 65 years old. The median follow-up duration is 17 months for the entire cohort, being 19 months for non-diabetic and 15 months for diabetic patients. Among the 3,182 participants, 1001 (31.5%) were early HCC, 503 (15.8%) were intermediate HCC, 1282 (40.3%) were advanced HCC, while 396 patients (12.4%) had terminal HCC at the time of diagnosis. Prevalence of DM was 25.2% as a whole. The respective DM prevalence in BCLC very early, early, intermediate, advanced, and terminal stages were 22.0%, 24.1%, 21.9%, 27.2%, and 27.1%, showing no statistically significant difference (Table 1; p = 0.081). However, a significant trend toward increasing prevalence of DM in more advanced BCLC stages were noted (p for trend = 0.048, Fig 1).

**Table 1:** Demographic, clinical, and staging information of the entire hepatocellular carcinoma cohort.

n = 3,182	BCLC Stage 0 (n = 265)	BCLC Stage A (n = 736)	BCLC Stage B (n = 503)	BCLC Stage C (n = 1282)	BCLC Stage D (n = 396)	*P* value
Age (years, mean ± SD)	63.3 ± 11.8	64.9 ± 11.6	64.2 ± 13.6	63.8 ± 13.6	66.5 ± 14.8	0.004
Male gender, n (%)	185 (69.8)	528 (71.9)	414 (82.3)	1002 (78.3)	310 (78.3)	<0.001
Diabetes mellitus, n (%)	58 (22)	177 (24.1)	110 (21.9)	346 (27.2)	107 (27.1)	0.081
ECOG, n (%)						<0.001
Performance status = 0	265 (100)	736 (100)	503 (100)	296 (23.1)	6 (1.5)	
Performance status = 1	0 (0)	0 (0)	0 (0)	680 (53.0)	24 (6.1)	
Performance status = 2	0 (0)	0 (0)	0 (0)	306 (23.9)	30 (7.6)	
Performance status = 3	0 (0)	0 (0)	0 (0)	0 (0)	225 (56.8)	
Performance status = 4	0 (0)	0 (0)	0 (0)	0 (0)	111 (28.0)	
Vascular invasion, n (%)	0 (0)	0 (0)	0 (0)	591 (46.1)	215 (54.3)	<0.001
Hepatitis B, n (%)	141 (53.2)	377 (51.2)	299 (59.4)	705 (55.0)	197 (49.7)	0.019
Hepatitis C, n (%)	105 (39.6)	290 (39.4)	124 (24.7)	341 (26.6)	114 (28.8)	0.001
Alcoholism, n (%)	28 (10.6)	98 (13.3)	67 (13.3)	298 (23.2)	91 (23.0)	<0.001
Laboratory values (mean ± SD)						
Albumin (g/dl)	4.0 ± 0.5	3.9 ± 0.5	3.9 ± 0.5	3.6 ± 0.6	3.0 ± 0.6	<0.001
Bilirubin (mg/dl)	0.86 ± 0.39	0.94 ± 0.62	0.94 ± 0.83	1.42 ± 2.16	4.15 ± 5.85	<0.001
INR (ratio)	1.04 ± 0.09	1.06 ± 0.12	1.04 ± 0.12	1.10 ± 0.14	1.27 ± 0.33	<0.001
AFP (ng/ml)	136 ± 411	733 ± 9959	6281 ± 31499	32184 ± 165949	58138 ± 135642	<0.001
Platelet (1000/mm^3^)	133.8 ± 64.3	137.5 ± 62.3	174.5 ± 85.6	186.5 ± 106.2	196.6 ± 119.6	<0.001
Na (mEq/l)	139.4 ± 3.2	139.9 ± 3.0	139.6 ± 2.8	137.8 ± 3.8	134.9 ± 5.2	<0.001
eGFR* (ml/min/1.73 m^2^)	76.0 ± 22.0	74.4 ± 24.1	75.4 ± 24.8	78.2 ± 36.5	68.2 ± 40.0	<0.001
Ascites, n (%)	8 (3)	46 (6.3)	22 (4.4)	388 (30.3)	279 (70.5)	<0.001
Variceal bleeding, n (%)	2 (0.8)	9 (1.2)	5 (1.0)	53 (4.1)	43 (10.9)	<0.001
Total tumor volume (ml)	2.3 ± 1.3	17.1 ± 15.4	337.9 ± 541.1	570.0 ± 864.9	670.8 ± 1051.0	<0.001
Median follow-up duration (month)^+^	45 (25–78)	37 (17–68)	27 (12–59)	9 (3–26)	2 (1–9)	<0.001
Child-Turcotte-Pugh class (A/B/C), n (%)	265/0/0 (100/0/0)	651/85/0 (88.5/11.5/0)	459/44/0 (91.3/8.7/0)	873/409/0 (68.1/31.9/0)	76/164/156 (19.2/41.4/39.4)	<0.001
MELD score (mean ± SD)	8.2 ± 2.3	8.8 ± 2.9	8.7 ± 2.7	9.9 ± 3.7	14.8 ± 6.6	<0.001
Treatment modalities (resection/ablation/TACE/systemic therapy/others), %	38/50/11/0/1	42/35/21/0/2	42/7/45/2/4	22/11/32/17/18	3/9/14/14/60	<0.001

* eGFR: estimated glomerular filtration rate, calculated by the four-variable modification of diet in renal disease (MDRD) equation.

^**+**^ Data were demonstrated as medians (interquartile range)

Abbreviations: BCLC, Barcelona Clínic Liver Cancer classification; ECOG, Eastern Cooperative Oncology Group scale; INR, international normalized ratio for prothrombin time; AFP, Alpha-fetoprotein; Na, plasma sodium level; MELD, Model for End-Stage Liver Disease.

Age, sex, performance status, existence of vascular invasion, HBV or HCV infection, alcoholism, presence of ascites, history of variceal bleeding showed significant differences between different BCLC stages at baseline. Parameters with gradual increment from BCLC stage 0 to stage D were serum bilirubin (p< 0.001), international normalized ratio (INR) (p< 0.001), serum alpha-fetoprotein (AFP) level (p< 0.001), platelet count (p< 0.001), and total tumor volume (p< 0.001). On the contrary, some parameters decreased in graded manner, including serum albumin (p< 0.001), serum sodium (p< 0.001), and estimated glomerular filtration rate (eGFR, p< 0.001).

### Survival analysis

**Fig 2:**
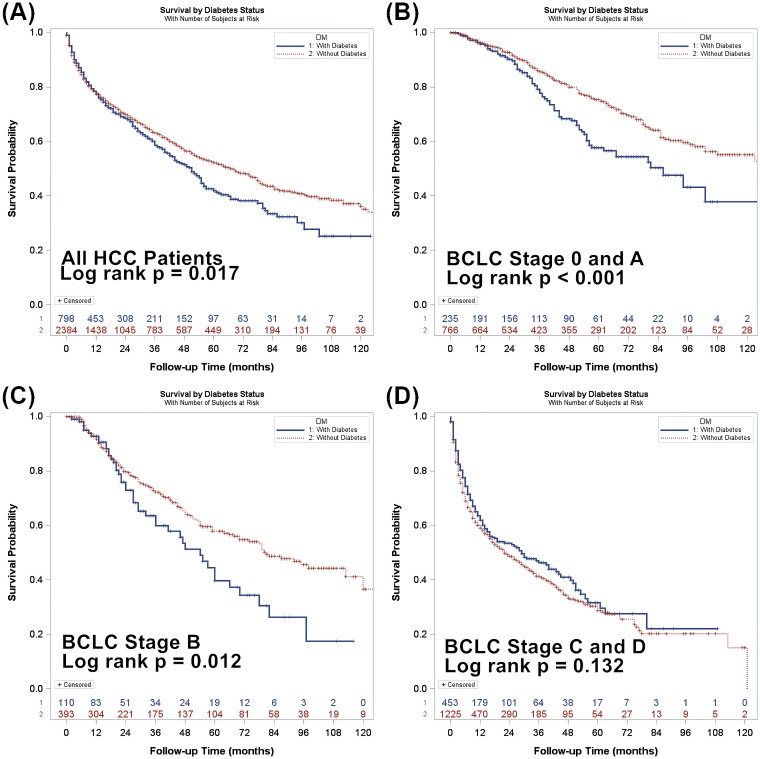
Cumulative survival rates of diabetic and non-diabetic patients among different BCLC staging groups. Differences in survival between diabetic and non-diabetic patients are significant in the whole cohort (p = 0.017, panel A), BCLC stage 0 and stage A (p< 0.001, panel B), and BCLC stage B (p = 0.012, panel C), but not in BCLC stage C and stage D (p = 0.132, panel D).

As a whole, diabetic HCC patients had significantly lower overall survival compared with non-diabetic patients (p = 0.017, Fig 2A). In subgroup analysis, diabetic patients also had decreased overall survival in very early/early and intermediate HCC (p<0.001 and 0.012, respectively, Fig 2B & 2C). The survival was similar between diabetic and non-diabetic patients in advanced and terminal HCC (p = 0.132, Fig 2D).

### Risk factor analysis

#### All patients

Univariate survival analysis show that DM, age, gender, HBV, alcoholism, albumin, bilirubin, INR, Na, AFP, eGFR, variceal bleeding, total tumor volume, vascular invasion, presence of ascites, and platelet count were significant predictors for survival in HCC patients (Table 2, all p< 0.05). Factors significant in the univariate analysis were introduced into the multivariate Cox model. MELD score and CTP class were not included in the final model because of they contain potentially confounding predictors, such as albumin and bilirubin. After adjustment, DM showed no effect on overall survival (adjusted hazard ratio [HR] 1.09; 95% confidence interval [CI] 0.95–1.25; p = 0.199).

**Table 2:** Univariate and multivariate Cox survival analysis in the entire hepatocellular carcinoma cohort.

n = 3,182	Crude Hazard Ratio		Adjusted Hazard Ratio	
	HR (95% CI)	*P* value	HR (95% CI)	*P* value
Diabetes mellitus (no/yes)	1.17 (1.03–1.33)	0.019	1.09 (0.95–1.25)	0.199
Age (per 10 years increment)	1.05 (1.00–1.10)	0.030	1.14 (1.09–1.20)	<0.001
Male gender (female/male)	1.16 (1.01–1.33)	0.039	1.07 (0.92–1.24)	0.370
Hepatitis B (negative/positive)	0.93 (0.83–1.04)	0.222	-	-
Hepatitis C (negative/positive)	0.84 (0.74–0.95)	0.007	0.97 (0.85–1.12)	0.702
Alcoholism (no/yes)	1.37 (1.19–1.59)	<0.001	1.16 (0.99–1.35)	0.064
Albumin (per 1 g/dl increment)	0.38 (0.35–0.42)	<0.001	0.55 (0.50–0.61)	<0.001
Bilirubin (per 1 mg/dl increment)	1.14 (1.13–1.15)	<0.001	1.07 (1.05–1.09)	<0.001
INR (per 1.0 increment)	4.80 (3.92–5.87)	<0.001	1.59 (1.18–2.13)	0.002
Sodium (per 1 mEq/l increment)	0.90 (0.89–0.92)	<0.001	0.98 (0.97–1.00)	0.029
AFP (per 10,000 ng/ml increment)	1.01 (1.01–1.01)	<0.001	1.01 (1.01–1.01)	<0.001
eGFR (per 10 mL/min/1.73 m^2^ increment)	0.96 (0.94–0.98)	<0.001	0.98 (0.96–0.99)	0.010
Variceal bleeding (no/yes)	2.17 (1.66–2.83)	<0.001	1.13 (0.85–1.49)	0.404
Total tumor volume (per 1,000 ml increment)	1.54 (1.48–1.61)	<0.001	1.22 (1.15–1.30)	<0.001
Vascular invasion (no/yes)	5.01 (4.42–5.68)	<0.001	3.20 (2.77–3.70)	<0.001
Ascites (no/yes)	3.52 (3.11–3.99)	<0.001	1.72 (1.48–1.99)	<0.001
Platelet count (per 10,000 mm^3^ increment)	1.03 (1.02–1.03)	<0.001	1.01 (1.01–1.02)	<0.001

Factors with *P* value < 0.1 in the univariate analysis were introduced into the Cox multivariate survival analysis.

The forepart of the parentheses was set as the reference group in univariate and multivariate analysis.

AFP, alpha-fetoprotein; CI, confidence interval; eGFR, estimated glomerular filtration rate; HR, hazard ratio; INR, international normalized ratio

#### BCLC stage 0 and stage A

DM, age, HBV, HCV, albumin, bilirubin, INR, AFP, eGFR, and platelet count showed significant impact on patient survival (Table 3, p< 0.05). After adjusting in the multivariate model, presence of DM (HR 1.45, 95% CI 1.08–1.93, p = 0.013), higher serum bilirubin (HR 1.27, 95% CI 1.07–1.52, p = 0.008), higher serum AFP (HR 1.34, 95% CI 1.20–1.48, p< 0.001), lower serum albumin (HR 1.82, 95% CI 1.37–2.38, p<0.001), and lower eGFR (HR 1.11, 95% CI 1.04–1.19, p = 0.001), lower platelet count (HR 1.03, 95% CI 1.0–1.05, p = 0.029) were confirmed as independent predictors of a decreased survival rate. We found no interaction between DM and these independent predictors (all p > 0.05).

**Table 3:** Univariate and multivariate Cox survival analysis in patients with BCLC stage 0 and stage A hepatocellular carcinoma.

n = 1,001	Crude Hazard Ratio		Adjusted Hazard Ratio	
	HR (95% CI)	*P* value	HR (95% CI)	*P* value
Diabetes mellitus (no/yes)	1.61 (1.23–2.12)	0.001	1.45 (1.08–1.93)	0.013
Age (per 10 years increment)	1.24 (1.11–1.38)	<0.001	1.11 (0.98–1.26)	0.089
Male gender (female/male)	1.02 (0.77–1.43)	0.902	-	-
Hepatitis B (negative/positive)	0.66 (0.52–0.85)	0.001	0.73 (0.52–1.01)	0.054
Hepatitis C (negative/positive)	1.37 (1.07–1.76)	0.012	0.87 (0.63–1.22)	0.424
Alcoholism (no/yes)	1.21 (0.84–1.74)	0.309	-	-
Albumin (per 1 g/dl decrement)	2.33 (1.85–2.94)	<0.001	1.82 (1.37–2.38)	<0.001
Bilirubin (per 1 mg/dl increment)	1.51 (1.29–1.76)	<0.001	1.27 (1.07–1.52)	0.008
INR (per 1.0 increment)	3.58 (1.61–7.97)	0.002	1.37 (0.43–4.30)	0.593
Sodium (per 1 mEq/l increment)	1.03 (0.99–1.07)	0.210	-	-
AFP (per 10,000 ng/ml increment)	1.30 (1.17–1.44)	<0.001	1.34 (1.20–1.48)	<0.001
eGFR (per 10 mL/min/1.73 m^2^ decrement)	1.12 (1.06–1.19)	<0.001	1.11 (1.04–1.19)	0.001
Variceal bleeding (no/yes)	1.39 (0.44–4.33)	0.574	-	-
Total tumor volume (per 1,000 ml increment)	1.05 (0.96–1.14)	0.274	-	-
Vascular invasion (no/yes)	-	-	-	-
Ascites (no/yes)	1.55 (0.90–2.65)	0.114	-	-
Platelet count (per 10,000 mm^3^ decrement)	1.05 (1.03–1.08)	<0.001	1.03 (1.0–1.05)	0.029

Factors with *P* value < 0.1 in the univariate analysis were introduced into the Cox multivariate survival analysis.

The forepart of the parentheses was set as the reference group in univariate and multivariate analysis.

AFP, alpha-fetoprotein; CI, confidence interval; eGFR, estimated glomerular filtration rate; HR, hazard ratio; INR, international normalized ratio

#### BCLC stage B

In univariate survival analysis for BCLC stage B subgroup, DM, albumin, total tumor volume were associated with survival (Table 4, p< 0.05). The Cox multivariate analysis revealed DM (HR 1.77, 95% CI 1.24–2.51, p = 0.002), serum albumin (HR 1.67, 95% CI 1.27–2.22, p< 0.001), and total tumor volume (HR 1.51, 95% CI 1.19–1.93, p = 0.001) as independent predictors of a poor outcome. There were no interaction between DM and these independent predictors of poor outcome (all p > 0.05).

**Table 4:** Univariate and multivariate Cox survival analysis in patients with BCLC stage B hepatocellular carcinoma.

n = 503	Crude Hazard Ratio		Adjusted Hazard Ratio	
	HR (95% CI)	*P* value	HR (95% CI)	*P* value
Diabetes mellitus (no/yes)	1.54 (1.09–2.17)	0.014	1.77 (1.24–2.51)	0.002
Age (per 10 years increment)	1.09 (0.97–1.22)	0.157	-	-
Male gender (female/male)	0.86 (0.59–1.28)	0.463	-	-
Hepatitis B (negative/positive)	1.00 (0.74–1.35)	0.992	-	-
Hepatitis C (negative/positive)	0.98 (0.69–1.39)	0.914	-	-
Alcoholism (no/yes)	1.29 (0.83–2.00)	0.260	-	-
Albumin (per 1 g/dl increment)	1.72 (1.30–1.22)	<0.001	1.67 (1.27–2.22)	<0.001
Bilirubin (per 1 mg/dl increment)	1.11 (0.99–1.24)	0.082	1.10 (0.97–1.25)	0.144
INR (per 1.0 increment)	2.24 (1.00–5.02)	0.051	2.36 (0.89–6.27)	0.085
Sodium (per 1 mEq/l increment)	0.97 (0.92–1.02)	0.207	-	-
AFP (per 10,000 ng/ml increment)	1.02 (0.97–1.07)	0.395	-	-
eGFR (per 10 mL/min/1.73 m^2^ increment)	0.98 (0.92–1.05)	0.595	-	-
Variceal bleeding (no/yes)	2.49 (0.62–10.08)	0.200	-	-
Total tumor volume (per 1,000 ml increment)	1.29(1.01–1.64)	0.041	1.51 (1.19–1.93)	0.001
Vascular invasion (no/yes)	-	-	-	-
Ascites (no/yes)	1.34 (0.68–2.62)	0.396	-	-
Platelet count (per 10,000 mm^3^ increment)	0.98 (0.96–1.00)	0.099	0.99 (0.97–1.01)	0.182

Factors with *P* value < 0.1 in the univariate analysis were introduced into the Cox multivariate survival analysis.

The forepart of the parentheses was set as the reference group in univariate and multivariate analysis.

AFP, alpha-fetoprotein; CI, confidence interval; eGFR, estimated glomerular filtration rate; HR, hazard ratio; INR, international normalized ratio

#### BCLC stage C and stage D

Variables showing hazardous effects for HCC survival were bilirubin (HR 1.11; 95% CI 1.09–1.12; p<0.001), INR (HR 3.13; 95% CI 2.46–3.97; p< 0.001), serum AFP (HR 1.01; 95% CI 1.01–1.01 p< 0.001), total tumor volume (HR per 1,000ml increment 1.33; 95% CI 1.26–1.40; p< 0.001), variceal bleeding (HR 1.38; 95% CI 1.04–1.83; p = 0.024), vascular invasion (HR 2.59; 95% CI 2.24–3.00; p< 0.001), ascites (HR 2.26; 95% CI 1.95–2.61; p< 0.001), and platelet count (HR per 10,000/m^3^ increment: 1.02; 95% CI 1.02–1.03; p< 0.001). DM does not appear to be a predictive variable for survival in this group of patients (crude HR 0.88; 95% CI 0.75–1.04; p = 0.136). Significant predictors for decreased survival rate in multivariate Cox model were serum albumin (HR 1.67; 95% CI 1.47–1.89; p< 0.001) and bilirubin (HR 1.06; 95% CI 1.04–1.08; p< 0.001), plasma sodium (HR 1.02; 95% CI 1.01–1.04; p = 0.006), serum AFP (HR per 10,000 ng/ml increment 1.01; 95% CI 1.00–1.01; p< 0.001), eGFR (HR 1.03, 95% CI 1.01–1.05; p = 0.003), total tumor volume (HR per 1,000 ml increment 1.12; 95% CI 1.04–1.20; p = 0.002), vascular invasion (HR 2.34; 95% CI 1.99–2.74; p< 0.001), ascites (HR 1.63; 95% CI 1.39–1.92; p< 0.001), and platelet count (HR per 10,000/mm^3^ increment 1.01; 95% CI 1.01–1.02; p< 0.001; Table 5).

**Table 5:** Univariate and multivariate Cox survival analysis in patients with BCLC stage C and stage D hepatocellular carcinoma.

n = 1,678	Crude Hazard Ratio		Adjusted Hazard Ratio	
	HR (95% CI)	*P* value	HR (95% CI)	*P* value
Diabetes mellitus (no/yes)	0.88 (0.75–1.04)	0.136	-	-
Age (per 10 years increment)	0.99 (0.94–1.04)	0.589	-	-
Male gender (female/male)	1.19 (0.99–1.42)	0.059	1.09 (0.90–1.30)	0.387
Hepatitis B (negative/positive)	1.04 (0.90–1.20)	0.582	-	-
Hepatitis C (negative/positive)	0.81 (0.68–0.96)	0.013	1.00 (0.83–1.19)	0.954
Alcoholism (no/yes)	1.08 (0.91–1.28)	0.371	-	-
Albumin (per 1 g/dl decrement)	2.08 (1.85–2.27)	<0.001	1.67 (1.47–1.89)	<0.001
Bilirubin (per 1 mg/dl increment)	1.11 (1.09–1.12)	<0.001	1.06 (1.04–1.08)	<0.001
INR (per 1.0 increment)	3.13 (2.46–3.97)	<0.001	1.22 (0.86–1.72)	0.260
Sodium (per 1 mEq/l decrement)	1.09 (1.06–1.10)	<0.001	1.02 (1.01–1.04)	0.006
AFP (per 10,000 ng/ml increment)	1.01 (1.01–1.01)	<0.001	1.01 (1.00–1.01)	<0.001
eGFR (per 10 mL/min/1.73 m^2^ decrement)	1.03 (1.01–1.05)	0.010	1.03 (1.01–1.05)	0.003
Variceal bleeding (no/yes)	1.38 (1.04–1.83)	0.024	1.00 (0.74–1.34)	0.983
Total tumor volume (per 1,000 ml increment)	1.33 (1.26–1.40)	<0.001	1.12 (1.04–1.20)	0.002
Vascular invasion (no/yes)	2.59 (2.24–3.00)	<0.001	2.34 (1.99–2.74)	<0.001
Ascites (no/yes)	2.26 (1.95–2.61)	<0.001	1.63 (1.39–1.92)	<0.001
Platelet count (per 10,000 mm^3^ increment)	1.02 (1.02–1.03)	<0.001	1.01 (1.01–1.02)	<0.001

Factors with *P* value < 0.1 in the univariate analysis were introduced into the Cox multivariate survival analysis.

The forepart of the parentheses was set as the reference group in univariate and multivariate analysis.

AFP, alpha-fetoprotein; CI, confidence interval; eGFR, estimated glomerular filtration rate; HR, hazard ratio; INR, international normalized ratio

## Discussion

In this longitudinally followed-up study from a large patient cohort, notably, a quarter of HCC patients were diabetic. We demonstrate a trend toward increasing prevalence of DM in HCC patients with higher BCLC stages. In addition, DM may differentially affect overall survival in HCC patients with BCLC stage 0, stage A, and stage B subgroups, but not in those with BCLC stage C and D groups.

Relations between DM and HCC prognosis have been studied extensively but show discrepant results. It has been noticed in several epidemiologic studies that the predictive value of DM on HCC prognosis was limited to specific patient subgroups. Wang et al. reported a meta-analysis including 21 studies with total 9,767 HCC patients, and showed that DM is an independent predictor for decreased overall survival (HR 1.55; 95% CI 1.27–1.91; *p* = 0.001) and disease-free survival (HR 2.15; 95% CI 1.75–2.63; *p* = 0.001).[12] However, subgroup analyses in that study further disclosed that the effect was only seen in patients receiving hepatic resection (HR 1.91; 95% CI 1.21–3.00; *p* = 0.005), but not in subjects treated with other modalities.

Whether differences exist in tumors with diverse baseline characteristics is less well established. Our group previously conducted an analysis comparing how DM influenced cumulative survival in patients with small (defined as ≤ 5 cm) and large (> 5 cm) hepatic tumors. We found that non-diabetic patients had significantly better survival after surgical resection compared to diabetic patients when the tumors are small. In contrast, there was no significant difference in survival between DM and non-DM individuals when tumor sizes were large at presentation.[27]

HCC is a highly heterogeneous disease entity, and the size of tumor size does not necessarily correlate well with disease severity, tumor stage, or prognosis. The BCLC classification system involves not only tumor size, but also performance status and liver functional reserve. Thus it may serve as a better disease indicator. In the current study, we divided patients into a total of three subgroups according to their BCLC staging. Our aim is to more precisely evaluate how the presence of DM changes the prognosis of HCC under different disease entities. We found a poor overall survival in diabetic patients with early BCLC stages but not in those with advanced diseases. This phenomenon could be explained by the hepatocarcinogenesis effect of insulin. In type 2 diabetic patients, increased insulin resistance and resulting hyperinsulinemia might upregulate the production of insulin-like growth factor-1 (IGF-1) and insulin receptor subtrate-1 (IRS-1). Elevating IGF-1 stimulates cell proliferation and inhibits apoptosis, thereby inducing carcinogenesis.[28] Consequently, patients with DM may suffer from accelerating tumor growth and poorer survival. However, in chronic and advanced liver diseases, especially in fibrotic liver, insulin resistance increased.[29–32] We consider that in BCLC stage C and stage D populations, the underlying liver disease alone causes insulin resistance and hyperinsulinemia that resemble diabetes status. Therefore, whether or not patients have true clinical diabetes may not significantly influence the prognosis. This assumption was further supported by our demographic data showing that the CTP class was more advanced in higher BCLC stages, representing more severe cirrhosis. In the meanwhile, the same hypothesis could also explain the gradual increasing trend of DM prevalence from BCLC stage 0 to stage D as observed in our cohort, which stems from progressively worsening insulin resistance.

DM is known to cause multi-system complications. Diabetic patients with early stage HCC usually have a better survival, which allows diabetic complications and diabetes-associated death to develop. Thus the increased mortality in diabetic group might come from diabetic complications instead of cancer burden. Alternatively, in later stages, treatment for HCC is greatly limited. According to the suggestions from the AASLD guideline, patients with BCLC stage C disease can only be treated with sorafenib, and stage D patients are less likely to tolerate any treatment except for palliative care. There is a high possibility that patients die before significant diabetic complications take place.

The study has a few limitations. This is a single-center study and the results may not be generalizable to other geographical areas. With more than half of the patients having evidence of HBV infection, our data require validation from other study groups. Secondly, as a tertiary center, referral bias cannot be avoided completely. Thirdly, our primary outcome was all-cause mortality. We could not evaluate the association between DM and its potential morbidities, such as surgical complications and cardiovascular death. Lastly, our study lacks of the information about DM treatment. Recently, the protective effect of several classes of anti-diabetic agents in cancer development and progression, especially metformin and thiazolidinedione, has been emphasized.[33, 34] On the contrary, insulin analogue seems to have mitogenic effects.[35] Therefore, choice of glucose lowering drugs may have its own role in affecting the outcome of HCC patients.

In conclusion, DM is highly prevalent among patients with HCC across different cancer stages. DM worsens overall survival in these patients with early BCLC stages from stage 0 to B. However. For patients with stage C and stage D, the long-term survival is not significantly influenced by the presence of DM.

## Abbreviations

AASLD: American Association for the Study of Liver Diseases
ADA: American Diabetes Association
AFP: alpha-fetoprotein
BCLC: Barcelona Clínic Liver Cancer
CI: confidence interval
CTP: Child-Turcotte-Pugh classification
DM: diabetes mellitus
EASL: European Association for the Study of the Liver
ECOG: Eastern Cooperative Oncology Group
eGFR: estimated glomerular filtration rate
HBV: hepatitis B virus
HCC: hepatocellular carcinoma
HCV: hepatitis C virus
HR: hazard ratio
IGF-1: insulin-like growth factor-1
INR: international normalized ratio
IRS-1: insulin receptor subtrate-1
MELD: model for end-stage liver disease
